# DYRK1-mediated phosphorylation of endocytic components is required for extracellular lumen expansion in ascidian notochord

**DOI:** 10.1186/s40659-023-00422-9

**Published:** 2023-03-11

**Authors:** Xiuke Ouyang, Bingtong Wu, Haiyan Yu, Bo Dong

**Affiliations:** 1 Fang Zongxi Center, MoE Key Laboratory of Marine Genetics and Breeding, College of Marine Life Sciences, Ocean University of China, Qingdao, 266003 China; 2 Laoshan Laboratory, Qingdao, 266237 China; 3 Institute of Evolution & Marine Biodiversity, Ocean University of China, Qingdao, 266003 China

**Keywords:** Endocytosis, DYRK1, Phosphorylation, Lumen expansion, Biological tube

## Abstract

**Background:**

The biological tube is a basal biology structure distributed in all multicellular animals, from worms to humans, and has diverse biological functions. Formation of tubular system is crucial for embryogenesis and adult metabolism. Ascidian *Ciona* notochord lumen is an excellent in vivo model for tubulogenesis. Exocytosis has been known to be essential for tubular lumen formation and expansion. The roles of endocytosis in tubular lumen expansion remain largely unclear.

**Results:**

In this study, we first identified a dual specificity tyrosine-phosphorylation-regulated kinase 1 (DYRK1), the protein kinase, which was upregulated and required for ascidian notochord extracellular lumen expansion. We demonstrated that DYRK1 interacted with and phosphorylated one of the endocytic components endophilin at Ser263 that was essential for notochord lumen expansion. Moreover, through phosphoproteomic sequencing, we revealed that in addition to endophilin, the phosphorylation of other endocytic components was also regulated by DYRK1. The loss of function of DYRK1 disturbed endocytosis. Then, we demonstrated that clathrin-mediated endocytosis existed and was required for notochord lumen expansion. In the meantime, the results showed that the secretion of notochord cells is vigorous in the apical membrane.

**Conclusions:**

We found the co-existence of endocytosis and exocytosis activities in apical membrane during lumen formation and expansion in *Ciona* notochord. A novel signaling pathway is revealed that DYRK1 regulates the endocytosis by phosphorylation that is required for lumen expansion. Our finding thus indicates a dynamic balance between endocytosis and exocytosis is crucial to maintain apical membrane homeostasis that is essential for lumen growth and expansion in tubular organogenesis.

**Supplementary Information:**

The online version contains supplementary material available at 10.1186/s40659-023-00422-9.

## Background

Biological tubes are present in all multicellular animals, and serve diverse physiological functions including transportation of nutrients, waste and gases, and structural support. The formation of distinct types of tubes has been previously reported [1, 2]. Exocytosis plays a crucial role in tubular formation and expansion [3–5]. However, the role of endocytosis in tubular lumen expansion remains largely unclear.

Endocytosis is a primary vesicle transport mechanism in eukaryotes and most endocytic events are mediated by clathrin-mediated endocytosis (CME) [6]. After the initiation of endocytic events, the clathrin coat is first assembled at the endocytic site and promotes the membrane invagination [7–9]. Then, coated vesicles are separated from the donor membrane with the help of scission proteins. BAR domain proteins, such as endophilin, are crucial for the constriction and scission of the invagination neck by recruiting dynamin in this process [10, 11]. Finally, coated vesicles dissociate, and new vesicles fuse with the early endosome. Different mechanisms have been discovered for dissociation of endocytic complex. The heat shock cognate 71 kDa protein (HSC70) binds to clathrin through auxilin and promotes coat disassembly [12, 13]. Synaptojanin, a phosphoinositol phosphatase, promotes vesicle uncoating by dephosphorylating PI(4,5)P_2_ [14]. Phosphorylation also regulates the disassembly of clathrin-dependent endocytic machinery [15].

Dual specificity tyrosine-(Y)-phosphorylation-regulated kinase 1 (DYRK1) is a highly conserved serine–threonine protein kinase and a regulator of endocytosis by phosphorylating some endocytic components to promote endocytic complex disassembly in neuron development. DYRK1 phosphorylates dynamin1 and disturbs the interaction of dynamin1 with SH3 domain-containing proteins [16, 17]. Amphiphysin1 is phosphorylated by minibrain kinase, also known as DYRK1 kinase, to inhibit the interaction of amphiphysin1 with endophilin [18]. Minibrain-mediated phosphorylation is required for the interaction of synaptojanin with endocytic components and enhances synaptojanin enzyme activity [19, 20]. The uncoating process of clathrin-coated vesicles is promoted by DYRK1 by phosphorylating associated proteins, such as adaptin and AP180 [21]. Although DYRK1 was demonstrated to be involved in endocytosis, the conservation of mechanism that DYRK1 regulated endocytosis remain poorly understood in distinct species.

Ascidian *Ciona* notochord lumen is an excellent in vivo model for tubulogenesis. After convergence and intercalation, 40 notochord cells are aligned into a single row and extend along the anterior–posterior axis. Then, the extracellular lumen appears between adjacent notochord cells. After expansion and fusion, a single lumen was formed [22, 23]. Lumen expansion is a necessary process for tubular formation [24]. During lumen expansion, the apical membrane curve to the insides of the cell and surface area rapidly increases [1]. Lumen expansion is arrested by blocking vesicle trafficking [25]. Exocytotic vesicles are transported and fused with apical membrane depending on actin and microtubules distributed in the apical domain to promote membrane surface area increases [22, 23, 26].

In this study, we demonstrated that DYRK1 was required for ascidian notochord extracellular lumen expansion. We found that DYRK1 phosphorylated some key endocytic components which is vital for *Ciona* notochord lumen expansion. The loss of function of DYRK1 weakened endocytosis. Furthermore, we demonstrated that clathrin-mediated endocytosis (CME) existed and was required for notochord lumen expansion experimentally. We revealed that the exocytosis of notochord cells is vigorous in apical membrane. This suggests a dynamic balance between exocytosis and endocytosis exists to maintain membrane homeostasis during lumen expansion. Taken together, we thus propose a novel signaling pathway in which DYRK1 regulates the endocytosis by phosphorylation required for lumen expansion in *Ciona* notochord tubulogenesis.

## Results

### DYRK1 was upregulated and expressed in notochord during *Ciona* notochord lumen expansion

**Fig. 1:**
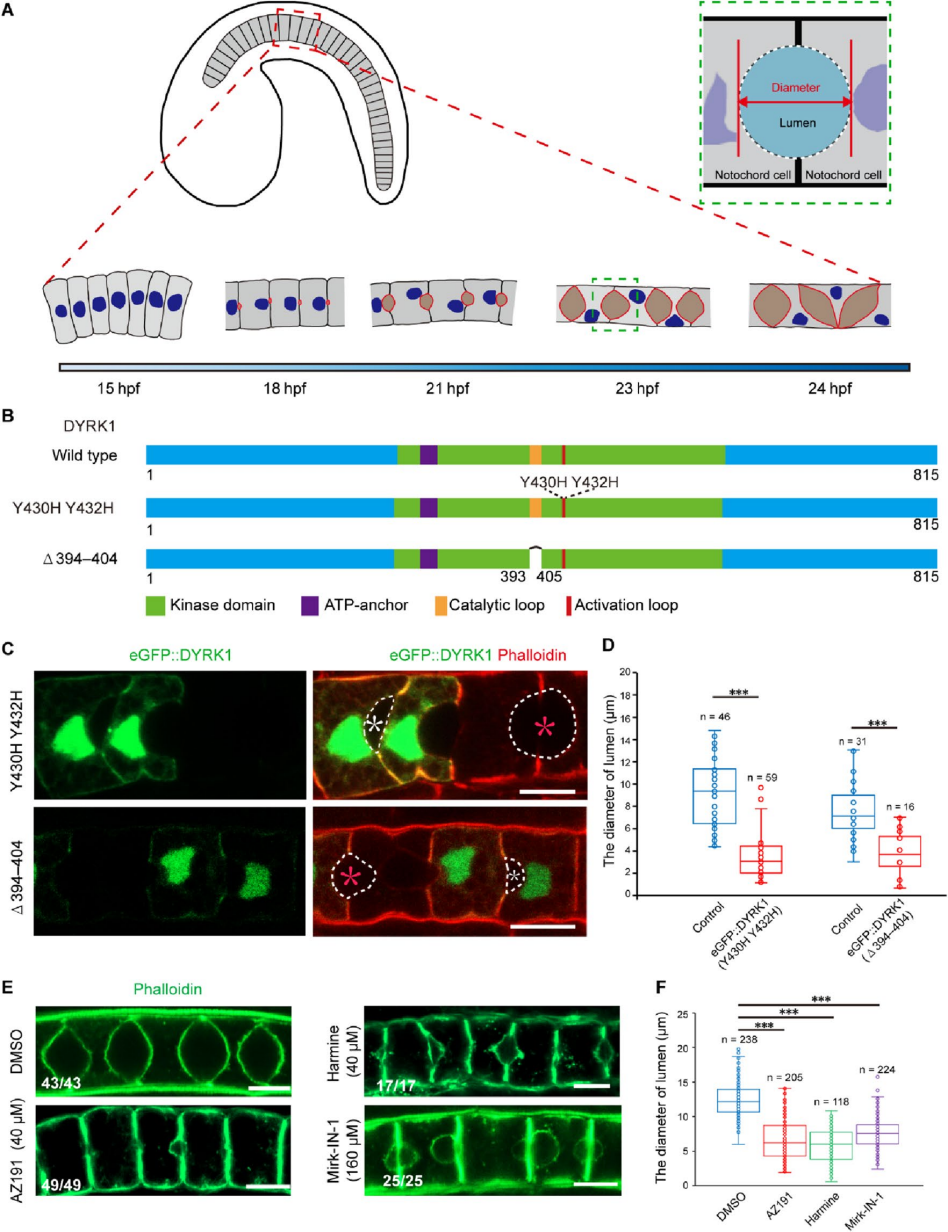
DYRK1-mediated phosphorylation is required for *Ciona* notochord lumen expansion. **A** The timeline of notochord tubulogenesis. The region of green dashed box was magnified to illustrate the spherical-lumen shape and the diameter of the lumen. **B** Schematic representation of the wild-type and mutant DYRK1 constructs. Kinase-inactive mutants were constructed by substituting tyrosine with histidine in the activation loop or deleting the catalytic loop (residues 394–404). **C** Overexpression of *DYRK1* mutants arrested lumen expansion. The white circle depicts the edge of the lumen, and the white asterisk indicates lumen with phenotype, and the red asterisk indicates control lumen. **D** The statistical analysis of lumen diameters. The “n” represents the number of lumens. Asterisks (***) represent statistical significance (p < 0.001). **E** Treatment with DYRK1 inhibitors (AZ191, 40 μM; Harmine, 40 μM; Mirk-IN-1, 160 μM) arrested lumen expansion. **F** The statistical analysis of lumen diameters. There was a significant difference in lumens diameter of control and the drug-treated groups. The “n” represents the number of lumens. Asterisks (***) represent statistical significance (p < 0.001). Scale bar 10 μm

*Ciona* notochord is composed of 40 rod-like cells, which elongate along the anterior–posterior axis during 15–18 h postfertilization (hpf). At approximately 18 hpf, an extracellular pocket lumen appeared between two adjacent notochord cells and expanded continuously from 18 to 24 hpf (Fig. 1A). Our previous work identified that *dual specificity tyrosine-(Y)-phosphorylation-regulated kinase 1* (*DYRK1*), a protein kinase, was upregulated during lumen expansion from 18 hpf (lumen initiation) to 21 hpf (lumen expansion) [27]. To verify this result, we performed qRT-PCR and confirmed that *DYRK1* had an expression peak during lumen expansion (Additional file 1: Figure S1A).

DYRK1 contains two homologs in mammals: DYRK1A and DYRK1B. However, only one homolog of DYRK1 was identified in the *Ciona* genome, suggesting that *DYRK1* was duplicated during metazoan evolution (Additional file 1: Figure S1B). It was shown in the phylogenetic tree that *Ciona* DYRK1 has a conserved kinase domain with DYRK subfamily mammal members.

To examine the tissue expression patterns of *DYRK1*, we made a construct containing 2974 bp upstream of *DYRK1* fused with eGFP (proDYRK1 > eGFP) and electroporated it into *Ciona* fertilized eggs. We found that *DYRK1* was mainly expressed in notochord (Additional file 1: Figure S1C). The anti-DYRK1 antibody immunofluorescence had a similar expression pattern. Close confocal observation revealed that DYRK1 was distributed on the apical membrane in a punctate form (Additional file 1: Figure S1D), suggesting that *DYRK1* engages in lumen formation and expansion.

### DYRK1-mediated phosphorylation is required for notochord lumen expansion

DYRK1 gains full enzyme activity by autophosphorylation on the second tyrosine of the activation loop and transfers the γ-phosphate of the ATP to substrate, depending on catalytic loop [28, 29]. Activation loop and catalytic loop were identified by multi-sequence alignment (Additional file 1: Figure S1E). To reveal the roles of DYRK1 phosphorylation in notochord lumen expansion, we made kinase-inactive mutants by substituting tyrosine with histidine in the activation loop or deletion of the catalytic loop (residues 394–404) (Fig. 1B). We forced them to be expressed in notochord by tissue-specific *brachyury* promoter (Fig. 1C). *Ciona* shows the classical mosaic gene expression pattern. When the plasmids were introduced into the embryos with a tissue-specific driver by electroporation, only partial of the tissue cells could express the plasmid. We therefore used those cells, which do not express plasmids as the internal control. The anteroposterior (A-P) diameter of spherical-lumen (Fig. 1A) in transgenic embryos was measured to evaluate the mutant phenotypes. The results showed that the diameter of lumen surrounded by DYRK1 mutant-overexpressed notochord cells was significantly shorter than additional pocket lumen served as control (Fig. 1D). Three different DYRK1 inhibitors (AZ191, 40 μM, Harmine, 40 μM, and Mirk-IN-1, 160 μM) were utilized to confirm the phenotypes further. These chemical inhibitors have a higher affinity with DYKR1 than ATP, inhibiting DYRK1 from catalyzing the substrates. The inhibition results showed that treatment with DYRK1 inhibitors arrested lumen expansion significantly (Fig. 1E–F). Together, DYRK1-mediated phosphorylation was demonstrated by these results to be required for notochord lumen expansion.

### Functional enrichment analysis of differentially phosphorylated proteins

To explore the substrates of DYRK1, 4D Label-free Quantitative Phosphoproteomics was applied to phosphorylation sequencing. The samples were collected after the DYRK1 inhibitor AZ191 and DMSO treatments. Pairwise Pearson’s correlation coefficients of all six samples (three replicates × two groups) showed high reproducibility (R > 0.9) of the phosphoproteomics data (Additional file 2: Figure S2A). A total of 14,913 phosphopeptides contained 8038 phosphosites corresponding to 2603 phosphoproteins (Additional file 2: Figure S2B). A total of 1556 differential phosphosites were screened (fold change  > 2 or < 0.5 and a p-value < 0.05), phosphorylation levels of 638 were significantly upregulated, and 918 were significantly downregulated (Additional file 2: Figure S2C, Additional file 5: Table S2). These phosphorylation level down-regulated proteins (PDPs) were further analyzed due to DYRK1 inhibition treatment. These proteins were shown with Gene Ontology (GO) enrichment analysis to be mainly involved in transport and localization (e.g., intracellular protein transport, protein transport, peptide transport, protein localization, and cellular protein localization) (Additional file 2: Figure S2D). Protein transport and localization largely depend on vesicle trafficking, which has been demonstrated to be essential in *Ciona* notochord lumen expansion [23, 25]. It was suggested by our phosphorylation sequencing results that lumen expansion is regulated by DYRK1 via the vesicle transport pathway.

### DYRK1 interacts with and phosphorylates endophilin

**Fig. 2:**
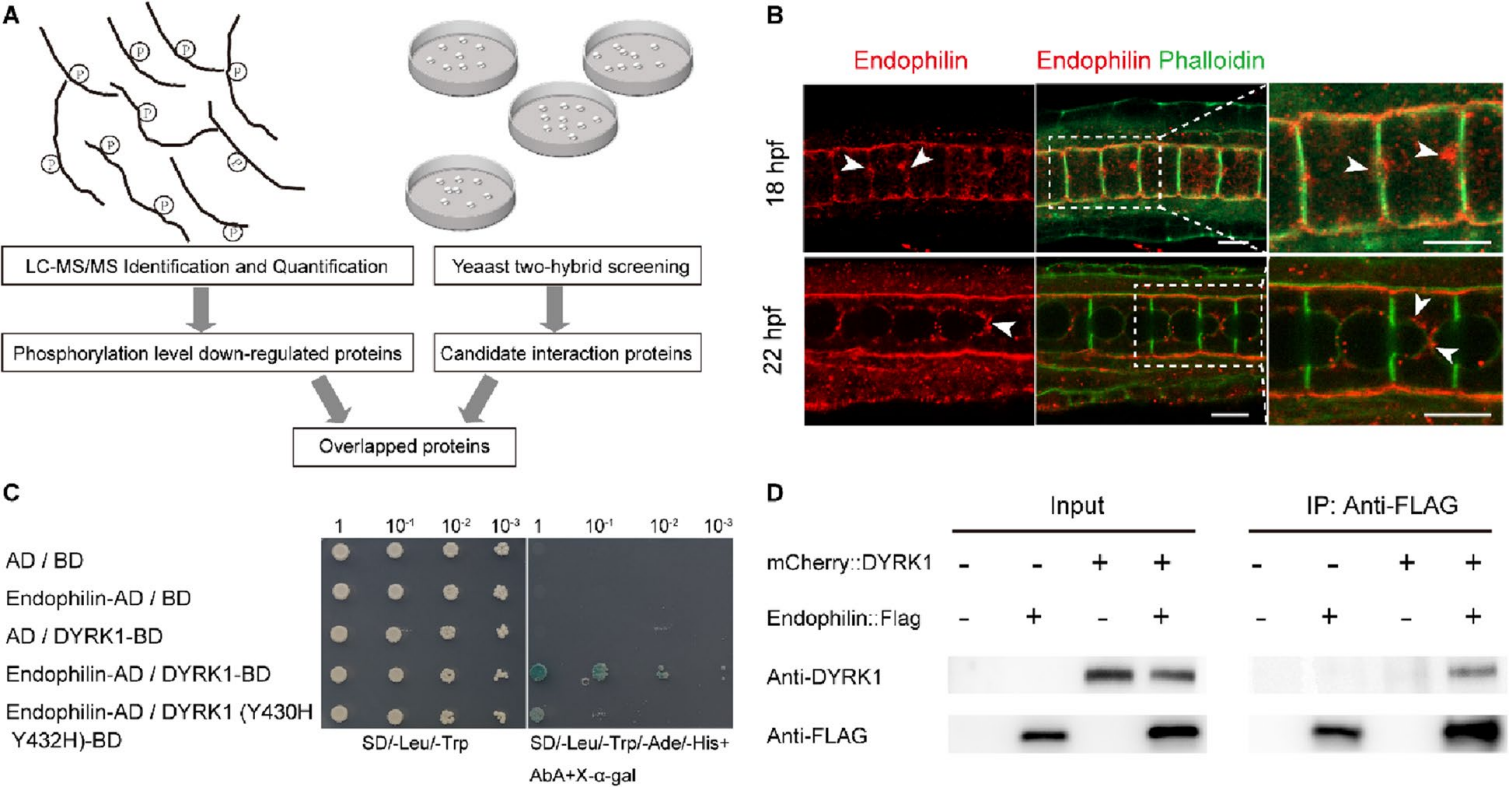
DYRK1 bound with and phosphorylated endophilin. **A** The workflow for screening DYRK1 substrates. Phosphoproteomics sequencing of DYRK1 inhibitor-treated samples identified the downregulated phosphorylation proteins. The potentially interacting proteins of DYRK1 were screened by a Y2H screening assay. Endophilin is a member of overlapped proteins by two technique approaches. **B** Subcellular localization of endophilin. It was shown by immunofluorescence that the endophilin was localized at the center of the anterior and posterior membrane regions where the lumen will appear (white arrowheads) at 18 hpf (the initiation of lumen formation). At 22 hpf (lumen expansion), endophilin was mainly enriched in the apical membrane (white arrowheads). The region of white box was magnified for observing localization. **C** Interaction assays between DYRK1 and endophilin by Y2H. Both kinase-active and inactive forms of DYRK1 interacted with endophilin. **D** Interaction of DYRK1 with endophilin was confirmed by Co-IP assay in the HEK293T cell line. Scale bar 10 μm

To further identify the direct substrates of DYRK1, yeast two-hybrid (Y2H) screening assay was performed. In total, 173 proteins were captured. We determined the intersection of the sets of the significantly downregulated phosphoproteins in phosphoproteomics and the proteins that interacted with DYRK1 in Y2H screening assay. Endophilin, an endocytic component, was a member of overlapped proteins by two technique approaches (Fig. 2A), suggesting that it is a potential DYRK1 substrate.

To explore the tissue expression pattern of *endophilin* in *Ciona*, immunofluorescence assay was performed. It was shown in the results that endophilin localized at the center of the anterior and posterior lateral membrane region, where the lumen will appear at 18 hpf (the initiation of lumen formation). At 22 hpf, endophilin was mainly enriched in the apical membrane (Fig. 2B), suggesting that endophilin engages lumen formation and lumen expansion. To identify the interaction of DYRK1 with endophilin, Y2H and coimmunoprecipitation assays were performed. It was shown in the result that DYRK1 interacted with wild-type endophilin and the N-terminus (residues 1–262) of endophilin but not the C-terminus (residues 263–356). Moreover, we found that the inactive form of DYRK1 interacted with endophilin (Fig. 2C–D).

Phosphoprotemic data showed that Ser263 phosphorylation of endophilin was regulated by DYRK1. This indicates the binding of DYRK1 with endophilin does not rely on phosphorylation. The N-terminus (residues 1–262) of endophilin contains a BAR domain implicated in protein dimerization and membrane curvature [30, 31]. Next, we found that individual BAR domains (residues 29–257) did not interact with DYRK1. Further results proved that residues 1–28 were necessary for this interaction, but not residues 258–262 (Additional file 3: Figure S3A). DYRK1 and endophilin had obvious colocalization in the notochord cell membrane and cytoplasm (Additional file 3: Figure S3B–C). Collectively, endophilin was suggested by these results to interact with DYRK1 and enriched in the apical membrane, being a potential regulator of notochord lumen formation and expansion.

### DYRK1-mediated phosphorylation of endophilin is required for lumen expansion

**Fig. 3:**
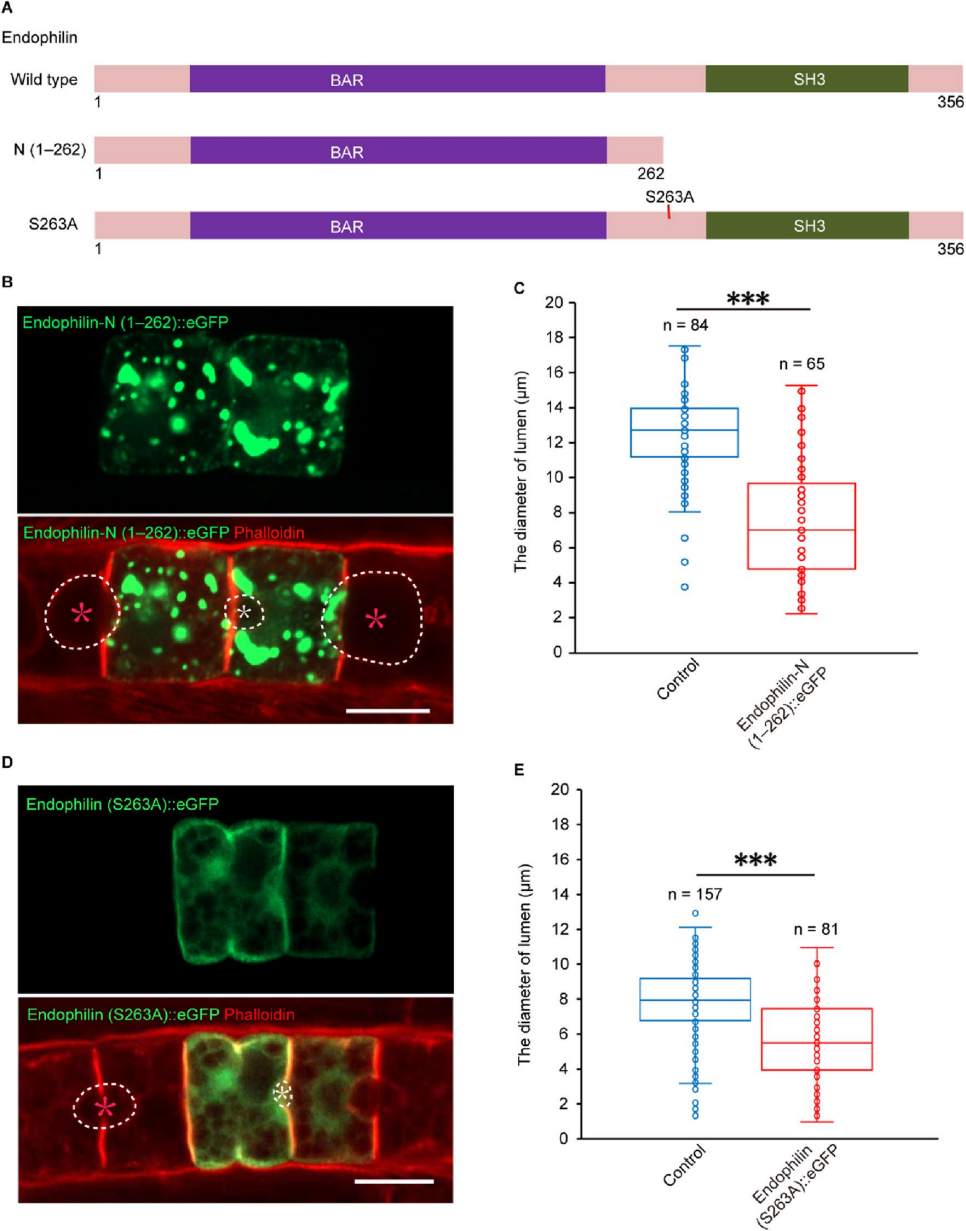
DYRK1-mediated phosphorylation of endophilin is required for *Ciona* notochord lumen expansion. **A** Schematic representation of the mutant constructs of endophilin (deletion of residues 263–356 or amino acid substitution at S263A). **B**–**C** Overexpression of endophilin-N (1–262) arrested lumen expansion significantly. **D**–**E** Overexpression of endophilin (S263A) arrested lumen expansion significantly. The white circle depicts the edge of the lumen, and the white asterisk indicates lumen with phenotype, and the red asterisk indicates control lumen. “n” represents the statistical number of the lumens. Asterisks (***) represent statistical significance (*p* < 0.001). Scale bar 10 μm

To explore the role of endophilin in lumen expansion, we made truncated endophilin-N (1–262), which served as a dominant negative [30, 31] (Fig. 3A) and forced it to be expressed in notochord (Fig. 3B). It was shown in the result that the diameter of the lumen surrounded by endophilin-N (1–262)-overexpressed notochord cells was significantly shorter than that in control (Fig. 3C). Overexpressing the phosphosite mutant endophilin (S263A) version (Fig. 3A) led to the failure of lumen expansion (Fig. 3D–E), phenocopying with loss of function of endophilin and DYRK1. It was indicated by these results that DYRK1-mediated phosphorylation of endophilin was required for notochord lumen expansion.

### DYRK1 is a regulator of endocytosis

**Fig. 4:**
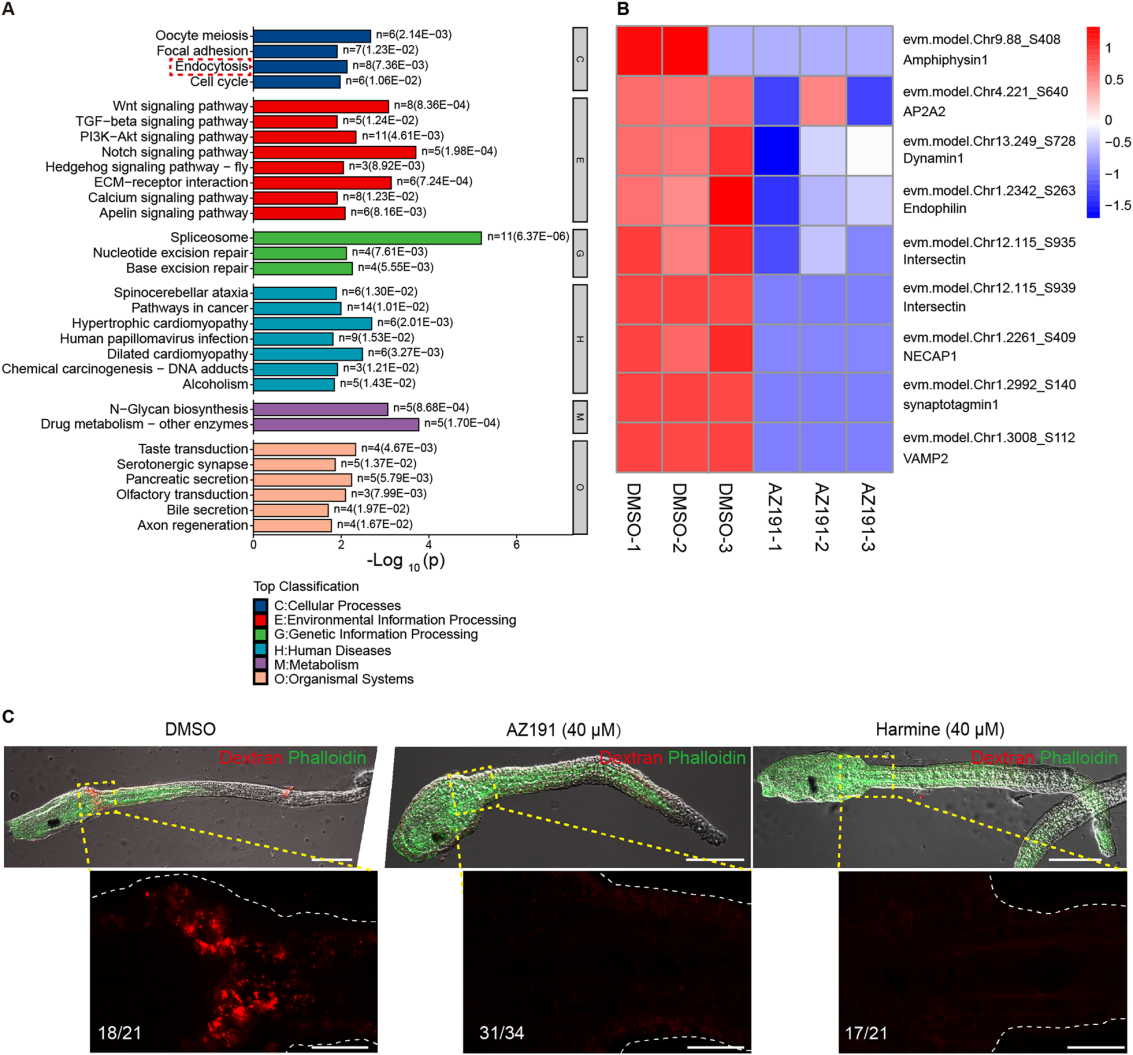
DYRK1 is an endocytosis regulator. **A** KEGG analysis of the phosphorylation level of the downregulated phosphoproteins. The significantly enriched 30 high-ranking KEGG pathways indicated that endocytosis was regulated by DYRK1. **B** The phosphorylation levels of some critical endocytic components and exocytotic components were downregulated. **C** The loss of function of DYRK1 by chemical inhibitors disturbed endocytosis. The labeled dextran in a punctate pattern was obviously observed in DMSO treatment embryos, while a relatively weak signal was found in DYRK1 inhibitor treatment embryos (AZ191, 40 μM; Harmine, 40 μM). A white dotted-line depicts edges of the magnified embryos. Scale bar in whole embryo panel, 100 μm; scale bar in magnification panel, 20 μm

Endophilin is essential for clathrin-mediated endocytosis (CME) by recruiting GTPase dynamin for the fission of coated pits and the phosphoinositide phosphatase synaptojanin for uncoating the coated vesicle [11, 32–34]. Except for endophilin, it was shown in the Kyoto Encyclopedia of Genes and Genomes (KEGG) analysis that the phosphorylation of many endocytic components was regulated by DYRK1 [e.g., amphiphysin1 (Ser408), NECAP1 (Ser409), dynamin1 (Ser728), AP2A2 (Ser640), and intersectin (Ser935, Ser939)] (Fig. 4A–B). Therefore, the essential regulation of DYRK1 phosphorylation on endocytosis in *Ciona* is suggested by these results. We performed a dextran dye uptake assay using *Ciona* embryos to determine whether DYRK1 engages in endocytic trafficking. The labeled dextran in a punctate pattern was observed in DMSO treatment embryos, while a relatively weak signal was observed in DYRK1 inhibitor treatment embryos (AZ191, 40 μM; Harmine, 40 μM) (Fig. 4C). This indicates that the endocytosis events in *Ciona* are regulated by DYRK1.

### Clathrin-mediated endocytosis is required for notochord lumen expansion

**Fig. 5:**
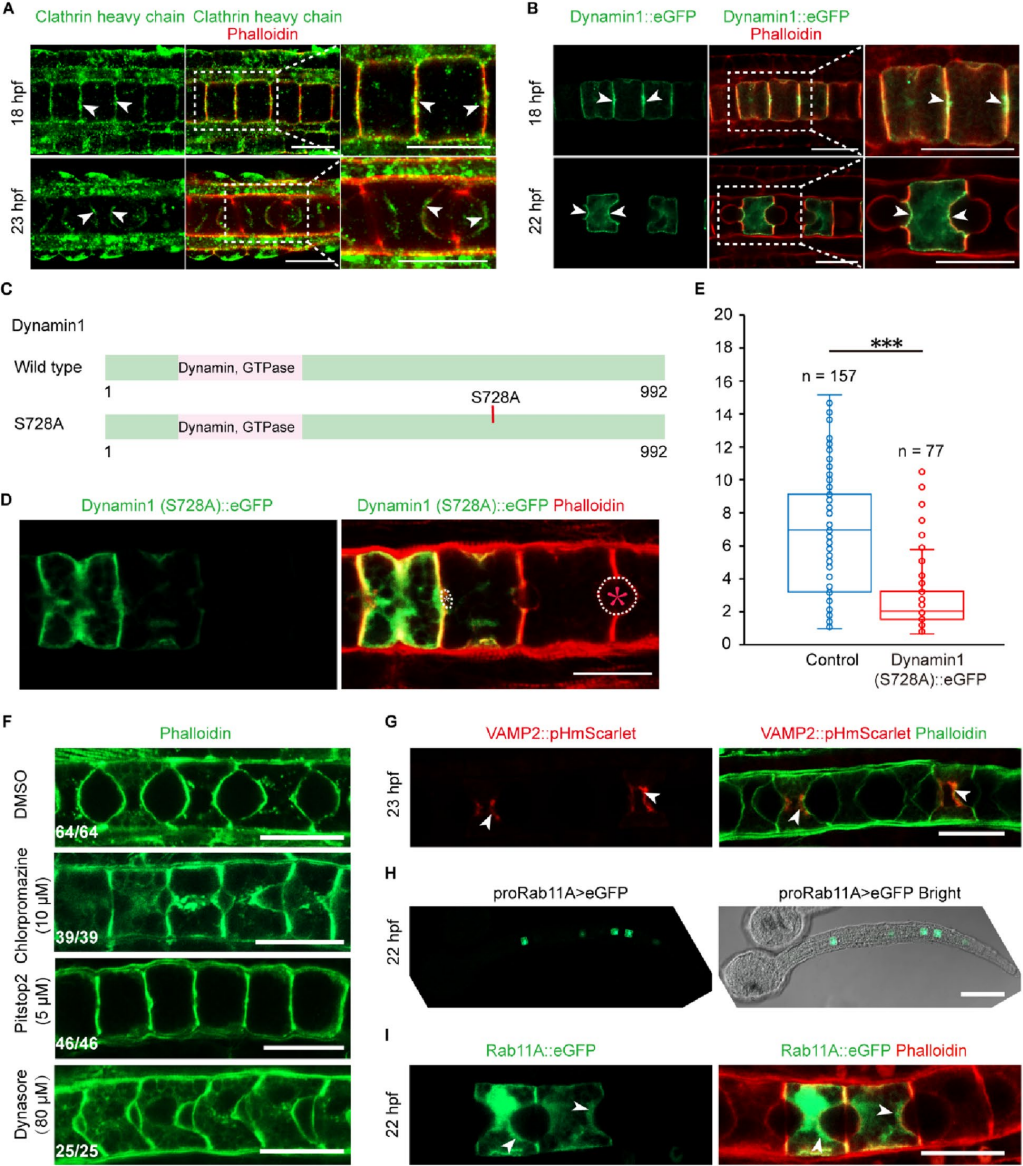
Clathrin-mediated endocytosis (CME) is required for expansion in *Ciona* notochord. **A**–**B** Both clathrin and dynamin 1, the main components of endocytosis, are localized at the center of the anterior and posterior membrane at the initiation of lumen formation. Both were mainly enriched in the apical membrane during lumen expansion. **C** Schematic representation of the mutant constructs of dynamin1 (S728A). **D**–**E** Overexpression of dynamin1 (S728A) arrested lumen expansion significantly. The white circle depicts the edge of the lumen, and the white asterisk indicates lumen with phenotype, and the red asterisk indicates control lumen. “n” represents the statistical number of lumens. Asterisks (***) represent statistical significance (*p* < 0.001). **F** Treatment with endocytosis inhibitors (Chlorpromazine, 10 μM; Pitstop2, 5 μM; Dynasore, 80 μM) arrested lumen expansion. **G** Exocytosis markers present in the apical membrane during notochord lumen expansion. pHmScarlet, a fluorescent protein, fused with VAMP2. The result showed VAMP2::pHmScarlet localized at the notochord apical membrane at 23 hpf. **H**–**I** Rab11A, the apical secreted vesicle coat protein, was expressed in notochord (promoter assay) **H** and localized at the apical membrane during notochord lumen expansion (**I**). Bars in A–B, D, F–G, and I are 20 μm. The bar in H is 100 μm

It is suggested by the above results that CME may be indispensable for notochord lumen expansion. Then, we first investigated the existence of CME in notochord cells. It was shown in the immunofluorescence assay of the clathrin heavy chain that the clathrin localized at the center of the anterior and posterior lateral membrane region where the lumen will appear. At 22 hpf, clathrin was mainly enriched in the apical membrane (Fig. 5A). Dynamin1, another endocytic component, also had a similar distribution to clathrin in the notochord (Fig. 5B). The distribution of endocytic components provided the cues that CME existed in the notochord cells. DYRK1-mediated phosphorylation of dynamin1 promotes endocytic apparatus disassociation [16]. Phosphoproteomics data showed DYRK1 phosphorylated dynamin1 on Serine728 (Fig. 4B). We made a phosphosite mutant construct that was expressed in notochord (Fig. 5C). The result showed that the diameter of the lumen surrounded by dynamin1 (S728A)-overexpressed notochord cells was significantly shorter than that in control (Fig. 5E). Furthermore, we used three different CME inhibitors (Chlorpromazine, 10 μM; Pitstop2, 5 μM; Dynasore, 80 μM) to treat *Ciona* embryos. The result showed that treatment with CME inhibitors prevented lumen expansion (Fig. 5F). Taken together, these results indicate CME was indicated by these results to be required for lumen expansion in *Ciona* notochord.

### The dynamic balance between exocytosis and endocytosis may occur in the apical membrane

Vesicle trafficking is necessary for notochord lumen expansion [23, 25]. However, the docking and fusion of exocytic vesicles with apical membrane has not been visualized in *Ciona* notochord cells. The vesicle-specific membrane protein VAMP2 (vesicle-associated membrane protein 2) was fused with fluorescent protein, pHmScarlet [35], for monitoring vesicle docking and the fusion of exocytosis, and the result showed that the fusion protein localized at the notochord apical membrane (Fig. 5G). The phosphoproteomics data showed that VAMP2 was a potential substrate of DYRK1 and was phosphorylated at Ser112 (Fig. 4B). Rab11A, a Rab11 family of small GTPases, is required for vesicle secretion and recycling [3, 36–38]. We found that *Rab11A* was specifically expressed in notochord and localized at the apical membrane during lumen expansion (Fig. 5H–I). The localization of these exocytosis markers showed that the exocytosis of notochord cells is vigorous in the apical membrane.

**Fig. 6:**
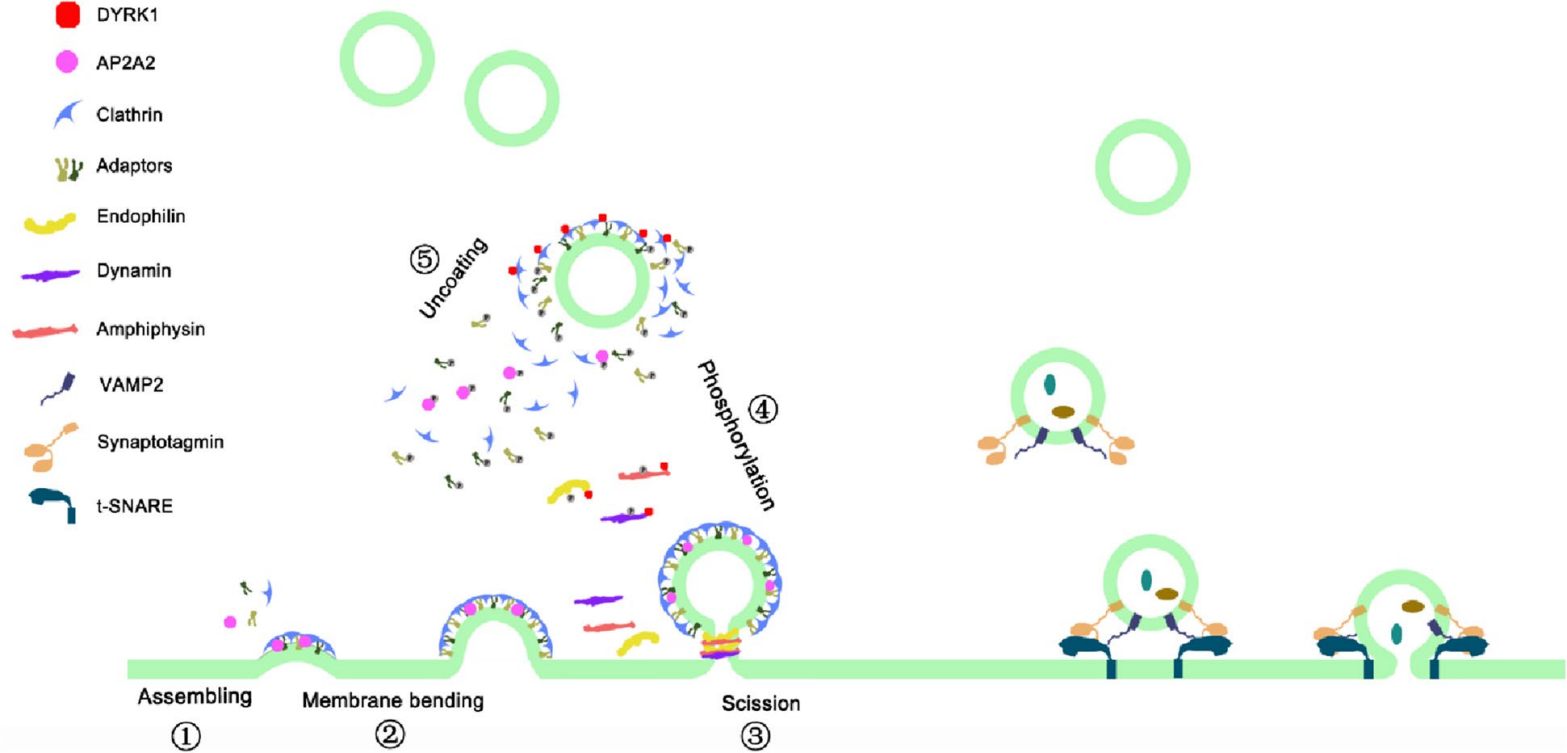
A potential working model for the role of endocytosis in *Ciona* lumen expansion

Our previous data in this study already showed that endocytosis also occurred during lumen expansion. Thus, we demonstrated the coexistence of endocytosis and exocytosis in the notochord apical membrane during lumen expansion. Both events might form a proper vesicle recycling mechanism to maintain the dynamic balance of membrane biogenesis. We propose a potential working model about the role of endocytosis in *Ciona* lumen expansion. DYRK1-mediated phosphorylation of endocytic components (e.g., dynamin1, endophilin, etc.) are required for endocytosis, which plays a crucial role in lumen expansion. Endocytosis and exocytosis co-exist in apical membrane of notochord cells, indicating that the dynamic balance of vesicle trafficking is essential for membrane biogenesis and lumen expansion (Fig. 6).

## Discussion

Endocytosis is a major vesicle transport mechanism in eukaryotes, and most endocytic events are mediated by CME [6]. This transport is required for many physiological processes in eukaryotic cells, such as maintaining membrane homeostasis and regulating intercellular signaling [39, 40]. It was found in previous studies that several endocytic components form a pioneer module containing the adaptor proteins FCHO1/2, AP2, the scaffold proteins EPS15, and intersectin1/2 that initiate endocytosis [10, 41]. Then, a clathrin coat containing clathrin, clathrin-adaptor proteins, and scaffold proteins (intersectins) were assembled [9]. Clathrin is recruited to the assembling coat depending on AP2, which was regulated by ear-binding coat-associated protein 1 (NECAP1) [42]. With the help of some scission proteins, such as dynamin1, the coated vesicle is separated from the donor membrane. BAR domain proteins, such as endophilin and amphiphysin1, are crucial for constriction and scission of the invagination neck [10]. Finally, a coated vesicle dissociates, and a new vesicle fuses with the early endosome. Several vital molecules play a crucial role in disassembling the clathrin-dependent endocytic machinery, such as HSC70, auxilin, and synaptojanin [12–14]. It was shown in previous studies that DYRK1 is required for the disassembly of the endocytic complex. DYRK1 phosphorylates dynamin1 and amphiphysin1 separately to suppress the interaction with endophilin [16–18]. In this study, we found that the phosphorylation of dynamin1 and amphiphysin1 was also regulated by DYRK1 through phosphoproteomics data in *Ciona*. This indicates the conservative mechanism in *Ciona*. Apart from known substrates involved in endocytosis, we demonstrated that DYRK1 interacted with and phosphorylated endophilin on Ser263. We speculated that DYRK1-phosphorylated endophilin inhibits the interaction of endophilin with other endocytic components. We also found that other endocytic components (e.g., AP2A2, intersectin, and NECAP1) involved in initiating endocytic events may be potential substrates of DYRK1. We further demonstrated that endocytosis was arrested by function deficiency of DYRK1. Collectively, the phosphorylation of many endocytic components involved in different steps of endocytosis was regulated by DYRK1, suggesting that DYRK1 may be a master-regulator and implicated in multi-steps of endocytosis.

Vesicle trafficking is indispensable for tubulogenesis. Vesicles are delivered to and fuse to increase the apical membrane area in an in vitro 3D cell culture model [5]. The secretion of the extracellular matrix and other components is crucial for maintaining apical membrane growth and lumen elongation in *Drosophila* [43, 44]. Single lumen formation depends on Rab11-mediated trafficking in zebrafish [3]. Arresting the vesicle trafficking endoplasmic reticulum and the Golgi complex disturbs the tubular formation in *Ciona* [25]. In this study, we used a fluorescent protein fused with VAMP2 to observe the vesicle docking and fusion in *Ciona* notochord lumen expansion. We found Rab11A localized at the notochord apical membrane during lumen expansion. It showed the exocytosis of notochord cells is vigorous in apical membrane. Continuous vesicle secretion disturbs the intracellular membrane homeostasis. It may need a proper mechanism to maintain the dynamic balance of the membrane. After exocytosis, the plasma membrane can be rapidly internalized by endocytosis and is used to generate new vesicles in the synapse [32]. This suggests that endocytosis may be an effective membrane recycling approach in lumen expansion. We found that CME existed in the notochord apical domain and was required for lumen expansion in *Ciona*. Endocytosis is also vital for recycling the luminal protein serpentine and driving tracheal airway maturation in the *Drosophila* [45, 46]. This suggests that endocytosis serves as a conserved mechanism regulating distinct types of tubular formation. Thus, we demonstrated the coexistence of endocytosis and exocytosis in the notochord apical membrane during lumen expansion. We speculated that there is a dynamic balance between exocytosis and endocytosis to maintain membrane homeostasis and avoid excessive expansion of the apical membrane. The apical membrane surface area continuously increases during lumen expansion, so exocytosis may be more potent than endocytosis.

Various cellular processes are regulated by posttranslational modifications (PTM), and phosphorylation is one of the most widely studied PTM. However, the contribution of phosphorylation to tube formation remains largely unclear. In this study, we found that DYRK1-mediated phosphorylation was required for lumen expansion, indicating the importance of phosphorylation. GO enrichment of PDPs was mainly involved in protein transport and localization. Endocytosis is a primary vesicle transport mechanism in eukaryotes, and most endocytic events are mediated by CME [6]. Furthermore, we demonstrated that DYRK1 was a regulator of endocytosis and that CME was required for lumen expansion. This indicates that the lumen expansion regulated by DYRK1 mainly affects endocytosis. We speculated that the loss of function of DYRK1 weakens endocytosis and potentially disturbs intracellular membrane homeostasis. Then, vesicle secretion is prevented, eventually arresting lumen expansion. In the phosphoproteomics data, we found that exocytotic components VAMP2 (evm.model.Chr1.3008_S112) and synaptotagmin1 (evm.model.Chr1.2992_S140) were potential substrates of DYRK1. Synaptotagmin1, a major Ca^2+^ sensor, interacts with phosphatidylinositol 4,5-biphosphate (PIP2) and helps with vesicle docking on the target membrane [47, 48]. VAMP2 (v-SNARE) binds with t-SNARE, which promotes vesicle fusion [49, 50]. It suggests that exocytosis may also be regulated by DYRK1. Clathrin-coated vesicles (CCVs) also are formed at the trans-Golgi network (TGN) and are transported to endosome and subsequently delivered to the plasma membrane [51]. Some components (e.g., clathrin, dynamin, and amphiphysin1) play a crucial role in the assembly of CCVs at the TGN and the plasma membrane [52, 53]. There exists a possibility that DYRK1 is implicated in the assembly of CCVs at the TGN, thereby regulates the exocytosis. This pathway might be involved in regulating the homeostasis of the apical membrane in notochord tubulogenesis. In addition, the recycling cargo could be transported back from the plasma membrane/endosomes to the Golgi [51]. CCVs play a key role in this process [54], which suggests that DYRK1 is involved in the regulation of the retrograde transport.

## Conclusions

Together our work identified that phosphorylation of component proteins involved in clathrin-mediated endocytosis is regulated by DYRK1. We revealed that the loss of function of DYRK1 disturbed endocytosis that existed and was required for notochord lumen expansion. Our results demonstrated the co-existence of endocytosis and exocytosis in the notochord apical membrane surface during lumen expansion. Both events might form a vesicle recycling mechanism to regulate the homeostasis of membrane biogenesis that is required for lumen growth and expansion in tubular organogenesis.

## Materials and methods

### Animals and electroporation

Adult animals were collected from the eastern coast of Qingdao (Shandong, China). Eggs or sperm were acquired from different individuals. Five minutes after fertilization, fertilized eggs were dechorionated, electroporated, and cultured at 16 ºC. All embryos were cultured in filtered seawater. Dechorionation and electroporation were conducted as previously described with some modifications [55]. The fertilized eggs (5 min post fertilization) were immersed in seawater containing 1% sodium thioglycolate, 0.05% protease E and 32 µl of 10 N NaOH, for removing the chorion at room temperature (RT) by gently pipetting. Dechorionated eggs were washed five times with filter-fresh seawater and transferred into 1% agarose-coated plastic Petri dishes for electroporation. Plasmid DNA (40 µg in 80 µl), 0.77 M mannitol (420 µl), and dechorionated eggs (300 μL) were mixed in 0.4 cm cuvettes. The exponential pulse protocol was used with the following conditions: Voltage 50 V, Capacitance 1500 μF and Resistance ∞. After electroporation, the fertilized eggs were washed and cultured at 16 ºC. Transgenic embryos were fixed with 4% paraformaldehyde (PFA) at room temperature for 2 h. The embryos were washed three times with phosphate-buffered saline (PBS) containing 0.1% Triton X-100 (0.1% PBST).

### Staining

The fixed embryos were washed three times with PBS containing 0.1% Triton X-100 (0.1% PBST). Then, the fixed embryos were stained with phalloidin for 12 h at 4 ºC, finally washed three times with PBS containing 0.1% Triton X-100 (0.1% PBST). Alexa Fluor 488 (Invitrogen) was dissolved with 1.5 ml of methanol and diluted (1:300) to mark the cell boundary of embryos. TRITC phalloidin (YEASEN) also was diluted (1:300) to mark the cell boundary. DAPI was used in the dark to mark the cell nucleus. Embryos were imaged using a Nikon A1 confocal microscope and a Zeiss LSM900 confocal microscope.

### qRT-PCR

*Ciona* embryos were collected at different time points: 0 hpf (unfertilized eggs), 5.5 hpf, 10 hpf, 14 hpf, 18 hpf, 21 hpf, 31 hpf, 42 hpf. Total RNA was obtained with RNAiso Reagent (Takara). First-strand cDNA was synthesized using HiScript II Q RT SuperMix for qPCR (Vazyme). The qRT-PCR was performed using ChamQ SYBR Color qPCR Master Mix (Vazyme) on a Light Cycler 96 (Roche). The reaction condition was as follows: 95 ºC for 30 S, 40 cycles at 95 ºC for 10 s and 60 ºC for 30 s, 95 ºC for 15 s, 65 ºC for 60 s, 95 ºC for 15 s. The expression level of *DYRK1* was normalized using *Tublin* as a reference. The results were analyzed using the 2^−ΔΔCt^ method.

### Gene structure, phylogenetic analysis and sequence alignment

The genomic and coding sequence (CDS) of *DYRK1* were obtained from the Ghost Database (http://ghost.zool.kyoto-u.ac.jp/cgi-bin/gb2/gbrowse/kh/). Gene structure analysis was performed using GSDS-2.0 (http://gsds.gao-lab.org/). The amino acids sequence of the DYRK subfamily was collected from the NCBI. Multiple sequence alignment was performed using Clustal-X software. Phylogenetic analysis was conducted using MEGA-X software. The phylogenetic tree was built using the maximum likelihood method with 1000 bootstraps, and the JTT model was selected. To search for conserved motif of DYRK1 in distinct species, amino acids sequence alignment was performed by using DNAMAN software.

### Plasmid constructions

PCR was performed with the Phanta Max Super-Fidelity DNA Polymerase (Vazyme). DNA was purified using a GeneJET Gel Extraction Kit (Thermofisher). All DNA Fragments were ligated by the One Step Cloning Kit (Vazyme). The promoter was amplified from *Ciona* genomic, and CDS was amplified from *Ciona* cDNA. The 2974 bp DNA sequence upstream of *DYRK1* was amplified by PCR and subcloned into KpnI and BamHI restriction sites of the pEGFP-1 vector (Clontech). The 3990 bp DNA sequence upstream of *Rab11A* was amplified by PCR and subcloned into SacI and SalI restriction sites of the pEGFP-1 vector (Clontech).

DYRK1 (Y430H Y432H) and DYRK1 (Δresidues 394–404) were amplified and subcloned into SacI and BamHI restriction sites of the Brachyury > eGFP-C1 vector (laboratory storage). DYRK1 was amplified and subcloned into BamHI and SacI restriction sites of the pGEX-6P-1 vector (Addgene). DYRK1 was amplified and subcloned into EcoRI restriction sites of the pGBKT7 vector (Clontech). DYRK1 (Y430H Y432H) was obtained from Brachyury > eGFP::DYRK1 (Y430H Y432H) and subcloned into EcoRI restriction sites of the pGBKT7 vector (Clontech). DYRK1 and mCherry were obtained and subcloned into NheI and BamHI restriction sites of the pEGFP-C1 vector (laboratory storage). Endophilin was amplified and subcloned into EcoRI and SalI restriction sites of the pET-30a vector (Addgene). Endophilin was amplified and subcloned into EcoRI and BamHI restriction sites of the CMV > FLAG vector (laboratory storage). Endophilin and its mutants, except for endophilin (Δresidues 1–28, 258–262), were amplified separately and subcloned into EcoRI restriction sites of the pGADT7 vector (Clontech). Endophilin (Δresidues 1–28, 258–262) was amplified from endophilin (Δresidues 258–262) and subcloned into EcoRI restriction sites of the pGADT7 vector (Clontech). Endophilin, endophilin (residues 1–262) and dynamin1 were amplified separately and subcloned into EcoRI restriction sites of the Bra > eGFP-N1 vector (laboratory storage). Endophilin (S263A) and dynamin1 (S728A) were amplified separately and subcloned into BamHI restriction sites of the Brachyury > eGFP-N1 vector (laboratory storage). CHC (residues 830–1686) was amplified and subcloned into EcoRI and SalI restriction sites of the pGXE-6P-1 vector (laboratory storage). Rab11A was amplified and subcloned into EcoRI and BamHI restriction sites of the Brachyury > eGFP-N1 vector (laboratory storage). VAMP2 was amplified and subcloned into BamHI restriction sites of the pHmscarlet-1 vector (from Xu Pingyong’s laboratory) driven by notochord-specific promoter brachyury. All PCR primers are listed in Additional file 4: Table S1.

### Immunofluorescence

The DYRK1, endophilin, and clathrin heavy chain antibodies were obtained by immunizing mice (Jinan Pengyue Laboratory animal breeding company, KM mouse). Fixed embryos were washed with 0.1% PBST and blocked with 10% goat serum in 0.1% PBST for 1 h at room temperature. Primary antibody diluted in 10% goat serum (DYRK1,1:200; endophilin, 1:300; clathrin heavy chain, 1:300) was used for 16 h at 4 ºC. After washing three times for 20 min each time with 0.1% PBST, a secondary antibody diluted in 10% goat serum (Alexa Fluor^™^ 555 anti-mouse IgG, 1:300) was used for 16 h at 4 ºC. Finally, embryos were washed three times for 20 min each time with 0.1% PBST.

### Drug treatment

For the function analysis assay, DYRK1 inhibitors (AZ191, 40 μM; Harmine, 40 μM; Mirk-IN-1, 160 μM) or endocytosis inhibitors (Chlorpromazine, 10 μM; Pitstop2, 5 μM; Dynasore, 80 μM) were added separately at 17 hpf, and up to 23 hpf embryos were collected and stained with phalloidin for imaging. For the dextran dye uptake assay, DYRK1 inhibitor (AZ191, 40 μM; Harmine, 40 μM) was separately added at 16 hpf, and dextran was added at 18 hpf, and the whole embryo was bathed in a dye solution. Up to 26 hpf embryos were collected and fixed.

For the 4D Label-free Quantitative Phosphoproteomics assay, AZ191 and DMSO were separately added at 17 hpf, and up to 23 hpf embryos were collected and stored in liquid nitrogen for phosphorylation sequencing. AZ191, Mirk-IN-1, Chlorpromazine, Dynasore, and Pitstop2 were purchased from MedChemExpress, and Harmine was purchased from Abcam.

### Data statistics and colocalization analysis

The longest path, considered as diameter of lumen, across the lumen was measured using ImageJ (https://imagej.nih.gov/ij/download.html). Comparison analysis of lumen diameter was performed using one-way ANOVA. A p < 0.05 was considered to indicate the existence of a significant difference. * represents 0.01, p < 0.05. ** represents 0.001 < p < 0.01. *** represents p < 0.001. The colocalization of tdTomato::DYRK1 and endophilin::eGFP was analyzed with ImageJ. Each channel of the image was gained by splitting the channels. Then, red and green channels were stacked, and the selected region was analyzed by a plot profile. The data were exported for plotting charts.

### LC–MS/MS-based quantitative phosphoproteomics

LC–MS/MS-based Quantitative Phosphoproteomics mainly contains sample preparation, protein digestion, phosphopeptide preparation, LC–MS analysis, database searching and analysis, and bioinformatics analysis. This assay was conducted at Shanghai Bioprofile Technology Co., Ltd.

### Reagents

Ammonium bicarbonate, dithiothreitol (DTT), iodoacetamide, sodium carbonate and formic acid (FA) were purchased from Sigma-Aldrich. Urea and sodium dodecyl sulfate (SDS) were purchased from Bio-Rad. Acetonitrile (ACN) and water for nano-LC − MS/MS were purchased from J. T. Baker. Trypsin was purchased from Promega. All other chemical reagents were purchased with analytical grade.

### Sample preparation and protein digestion

Embryo samples were ground into powder using liquid nitrogen. Approximately 20 mg of powder was resuspended with 200 μL lysis buffer (4% SDS, 100 mM DTT, 150 mM Tris–HCl pH 8.0). These samples were boiled and further ultrasonicated. Undissolved cellular debris were removed by centrifugation at 16,000 × g for 15 min. The supernatant was collected and quantified using a Bicinchoninic Acid Assay Protein Assay Kit (Bio-Rad, USA). Digestion of the protein was performed according to the FASP procedure. The protein was transferred into a 1.5 mL Nanosep tube (Pall Corporation, MWCO 10 K) and centrifuge for 20 min at 14,000 × g at 25 ºC to remove the DTT. Then, 100 μL 0.05 M iodoacetamide in UA buffer was added to block reduced cysteine residues, and these samples were incubated for 20 min in darkness. Then, 200 µL buffer of ammonium bicarbonate (100 mM) was added to the beads in the filter cartridge, centrifuge for 15 min (or until the 200 µL buffer passed through the filter) at 14,000 × g at 25 ºC. Finally, 50–100 μL of 50 mM ammonium bicarbonate buffer with trypsin at the enzyme-to-protein ratio of 1:50 was added to the sample and incubated at 37 ºC for 20 h. The peptides were harvested by centrifugation and then acidified with 1% FA. Subsequently, the peptides were dried using a refrigerated CentriVap concentrator (Labconco, Kansas, MO, USA). Finally, the tryptic digested peptides were desalted with a C18 stage tip.

### Phosphopeptide preparation

For phosphopeptide enrichment, the High-Select^™^ TiO2 Phosphopeptide Enrichment kit (Thermo Scientific) was used for digested peptide mixtures from each sample. Briefly, the TiO_2_ spin tip was equilibrated with wash buffer and binding/equilibration buffer by concentration sequentially. The desalted peptides were suspended in 150 μL binding/equilibration buffer and applied to the TiO_2_ spin tip. Afterward, the TiO_2_ spin tips were washed twice with binding/equilibration buffer and washing buffer sequentially. Then, LC–MS grade water was used for washing once, and the phosphopeptides were eluted from the TiO2 spin tip using 50 μL elution buffer twice. The phosphopeptides of elution were dried immediately and suspended with 0.1% FA for further LC–MS/MS analysis.

### LC–MS analysis

The phosphopeptides were loaded onto the C18 reversed-phase column (15 cm long, 75 μm ID, 2 μm, Dr. Maisch GmbH, Ammerbuch, Germany) in buffer A (2% ACN and 0.1% FA) and separated with a linear gradient of buffer B (90% ACN and 0.1% FA) at a flow rate of 300 nL/min over 60 min. The linear gradient was set as follows: 0–2 min, linear gradient from 2 to 5% buffer B; 2–42 min, linear gradient from 5 to 20% buffer B; 42–50 min, linear gradient from 20 to 35% buffer B; 50–52 min, linear gradient from 35 to 90% buffer B; 52–60 min, buffer B maintained at 90%. For MS data acquisition, the timsTOF Pro (Bruker) was operated in PASEF mode. The full scans were recorded from 100 to 1700 m/z spanning from 0.6 to 1.6 Vs/cm^2^ in the mobility (1/K0) dimension. The MS method was set as follows: Ramp time 100 ms, accumulation time 2.0 ms, Lock Duty Cycle to 100%, Capillary Voltage 1,700 V, Dry Gas 3 l/min, Dry Temp 180 ºC. After one full MS, 10 PASEF MS/MS frames were performed on ion-mobility separated precursors, excluding singly charged ions, which are fully segregated in the mobility dimension, with a threshold and target intensity of 1750 and 15,000 counts. CID collision energy was set at 42 eV.

### Database searching and analysis

The MS data were analyzed for data interpretation and protein identification against the *Ciona* genome (Unpublished). The MS spectra were searched using MSFragger version 2.4 and FragPipe version 13.1, with mass calibration and parameter optimization enabled. Tryptic cleavage specificity was applied, along with variable methionine oxidation (M), variable protein N-terminal acetylation, variable phosphorylation on serine (S), threonine (T), and tyrosine (Y), and fixed carbamidomethyl cysteine modifications. The allowed peptide length and mass ranges were 6–50 residues and 500–5,000 Da, respectively. PeptideProphet and ProteinProphet in Philosopher (version 2.2.0; https://philosopher.nesvilab.org/) were used to filter all phosphosite, peptide-spectrum matches, peptides, and proteins with < 1% false discovery rate. Entries from decoy proteins were removed. Label-free quantification analysis was performed with IonQuant (version 1.1.0). Site quantitation analyses were filtered only for those phosphorylation sites confidently localized (class I, localization probability > 0.75).

### Bioinformatics analysis

Analyses of bioinformatics data were conducted with Perseus software, Microsoft Excel, and R statistical computing software. Expression data were grouped by hierarchical clustering according to the protein or site level. Information was extracted from UniProtKB/Swiss-Prot, KEGG, and GO to annotate the sequences. GO and KEGG enrichment analyses were conducted with Fisher’s exact test, and FDR correction for multiple testing was also performed. GO terms were grouped into three categories: biological process, molecular function, and cellular component. Enriched GO and KEGG pathways were nominally statistically significant at p < 0.05.

### Y2H assay, coimmunoprecipitation assay

The cDNA library of *Ciona* was applied for the Y2H screening assay. All reagents of Y2H were purchased from Clontech. Constructs of prey and bait were transformed into Y2H Gold (Clontech) for identifying interaction. The Y2H assay was conducted as previously described [56]. HEK293T cells were obtained from Guo Huarong’s laboratory (Ocean University of China, Qingdao, China) and were used for identifying interaction. Cell culture, protein extraction, and western blotting were carried out as described previously [57]. HEK293T cells were cultured with DMEM medium with 15% FBS, 100 U/mL penicillin, 100 mg/mL streptomycin, and were maintained in 5% CO2 at 37 ºC. Cells were subcultured for further experiments after they became 80–90% confluent. Transfection in HEK293T cells was performed using Lipofectamine 3000 Invitrogen (Thermo Fisher). HEK293T cells were passaged in 6-well plates with 0.5 × 10^6^ cells per well. Then, plasmids (2500 ng) were used to transfect cells. The protein was incubated with Anti-Flag magnetic beads (Bimake, 580030) for 12 h. After incubation, the beads were washed three times with lysis buffer, then directly boiled in 1 × SDS loading buffer, and subjected to immunoblot analysis. The following antibodies were used in this study: anti-Flag (TransGen, HT201-01, 1:3,000); anti-DYRK1 (1:2,000); anti-Mouse IgG (H + L); and HRP conjugate (TransGen, 1:5,000, HS201–01).

## Supplementary Information

**Additional file 1: Figure S1.** The expression patterns, phylogenetic tree, and sequence alignment of DYRK1. (A) The expression level of *DYRK1* at different development stages. (B) Phylogenetic tree analysis of DYRK subfamily. Phylogenetic tree was built according to conserved kinase domain of DYRK subfamily. DYRK1 contains 2 homologues in mammal: DYRK1A, DYRK1B. However, only one homologue of DYRK1 was identified in *Ciona*. (C) *DYRK1* expressed in the notochord and other tissues through promoter assays. The notochord cells are indicated by the white arrowhead. The red box region was magnified to show the detailed expression pattern of *DYRK1*. (D) DYRK1 localized at the apical membrane in notochord cells (white arrowhead). (E) Sequence alignment of DYRK1. Some critical motifs of DYRK1 were highly conserved in distinct species. Scale bars in C and D are 100 μm and 20 μm, respectively.

**Additional file 2: Figure S2.** Functional enrichment analysis of differentially down-regulated phosphoproteins. (A) The quality assessment of the phosphorylation sequencing result. The values of Pairwise Pearson's correlation coefficients, shown with the blue text, between samples of same treatment were greater than 0.9, which suggests the high reproducibility (R > 0.9) of the phosphoproteomics data. (B) Total number of phosphosites, phosphopeptides, and phosphoproteins detected in phosphoproteomics. (C) Volcano plot of the DMSO/AZ191 group. The standard of fold change > 2 or < 0.5 and a P value < 0.05 were established. (D) GO enrichment of downregulated phosphoproteins.

**Additional file 3: Figure S3.** The interaction of DYRK1 with endophilin. (A) Identification of the binding fragment of endophilin with DYRK1. These results showed that the 28 residues at N-terminal of endophilin were required for the interaction of endophilin with DYRK1. (B–C) Endophilin colocalized with DYRK1 in notochord cell membrane and cytoplasm. Plot Profile of ImageJ software was used for colocalization analysis of tdtomato and eGFP signals. The variation trend of fluorescence intensity along the horizontal direction of the white dash line box region in B was showed in C. The red and green lines in C represent the variation trend of tdtomato and eGFP fluorescence intensity, respectively. The trends of red and green curve basically coincident suggest the colocalization of tdtomato and eGFP signals on the cell membrane and in the cytoplasm. The X-axis of C refers to the length in the horizontal direction of white dash line box. Scale bar 20 μm.

**Additional file 4: Table S1.** Primer for vector construction

**Additional file 5: Table S2.** Differential phosphoproteins

## Data Availability

The Phosphoproteomics data have been deposited to the ProteomeXchange Consortium (http://proteomecentral.proteomexchange.org) via the iProX partner repository with the dataset identifier PXD039234.

## Abbreviations

DYRK1: Dual specificity tyrosine-(Y)-phosphorylation-regulated kinase 1
CME: Clathrin-mediated endocytosis
CCV: Clathrin-coated vesicle
PDPs: Phosphorylation level down-regulated proteins
0.1% PBST: PBS containing 0.1% Triton X-100.
FA: Formic acid
ACN: Acetonitrile
TGN: Trans-Golgi network
PM: Plasma membrane
