# Supplementary material for: DYRK1-mediated phosphorylation of endocytic components is required for extracellular lumen expansion in ascidian notochord
Source: Biol Res. 2023 Mar 11;56:10. doi: 10.1186/s40659-023-00422-9 (PMC10007804; doi:10.1186/s40659-023-00422-9)
Supplement: Supplementary file 1 — Additional file 1: Figure S1. The expression patterns, phylogenetic tree, and sequence alignment of DYRK1. (A) The expression level of DYRK1 at different development stages. (B) Phylogenetic tree analysis of DYRK subfamily. Phylogenetic tree was built according to conserved kinase domain of DYRK subfamily. DYRK1 contains 2 homologues in mammal: DYRK1A, DYRK1B. However, only one homologue of DYRK1 was identified in Ciona. (C) DYRK1 expressed in the notochord and other tissues through promoter assays. The notochord cells are indicated by the white arrowhead. The red box region was magnified to show the detailed expression pattern of DYRK1. (D) DYRK1 localized at the apical membrane in notochord cells (white arrowhead). (E) Sequence alignment of DYRK1. Some critical motifs of DYRK1 were highly conserved in distinct species. Scale bars in C and D are 100 μm and 20 μm, respectively. [file 40659_2023_422_MOESM1_ESM.docx]

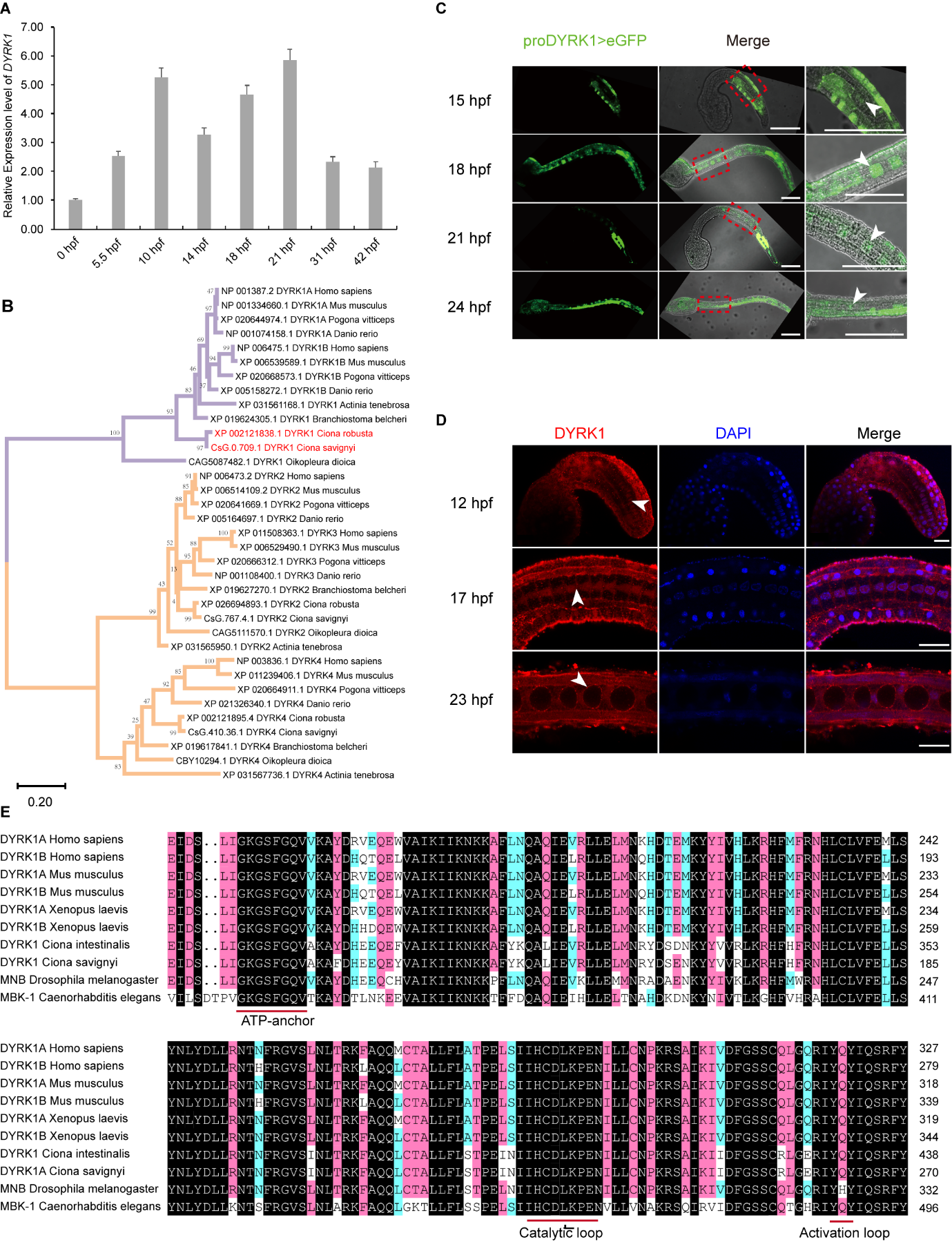


**Figure S1:** The expression patterns, phylogenetic tree and sequence alignment of DYRK1. (A) The expression level of *DYRK1* at different development stages. (B) Phylogenetic tree analysis of DYRK subfamily. Phylogenetic tree was built according to conserved kinase domain of DYRK subfamily. DYRK1 contains 2 homologues in mammal: DYRK1A, DYRK1B. However, only one homologue of DYRK1 was identified in *Ciona*. (C) *DYRK1* expressed in the notochord and other tissues through promoter assays. The notochord cells are indicated by the white arrowhead. The red box region was magnified to show the detailed expression pattern of *DYRK1*. (D) DYRK1 localized at the apical membrane in notochord cells (white arrowhead). (E) Sequence alignment of DYRK1. Some critical motifs of DYRK1 were highly conserved in distinct species. Scale bars in C and D are 100 μm and 20 μm, respectively.
