# Supplementary material for: DYRK1-mediated phosphorylation of endocytic components is required for extracellular lumen expansion in ascidian notochord
Source: Biol Res. 2023 Mar 11;56:10. doi: 10.1186/s40659-023-00422-9 (PMC10007804; doi:10.1186/s40659-023-00422-9)
Supplement: Supplementary file 2 — Additional file 2: Figure S2. Functional enrichment analysis of differentially down-regulated phosphoproteins. (A) The quality assessment of the phosphorylation sequencing result. The values of Pairwise Pearson's correlation coefficients, shown with the blue text, between samples of same treatment were greater than 0.9, which suggests the high reproducibility (R > 0.9) of the phosphoproteomics data. (B) Total number of phosphosites, phosphopeptides, and phosphoproteins detected in phosphoproteomics. (C) Volcano plot of the DMSO/AZ191 group. The standard of fold change > 2 or < 0.5 and a P value < 0.05 were established. (D) GO enrichment of downregulated phosphoproteins. [file 40659_2023_422_MOESM2_ESM.docx]

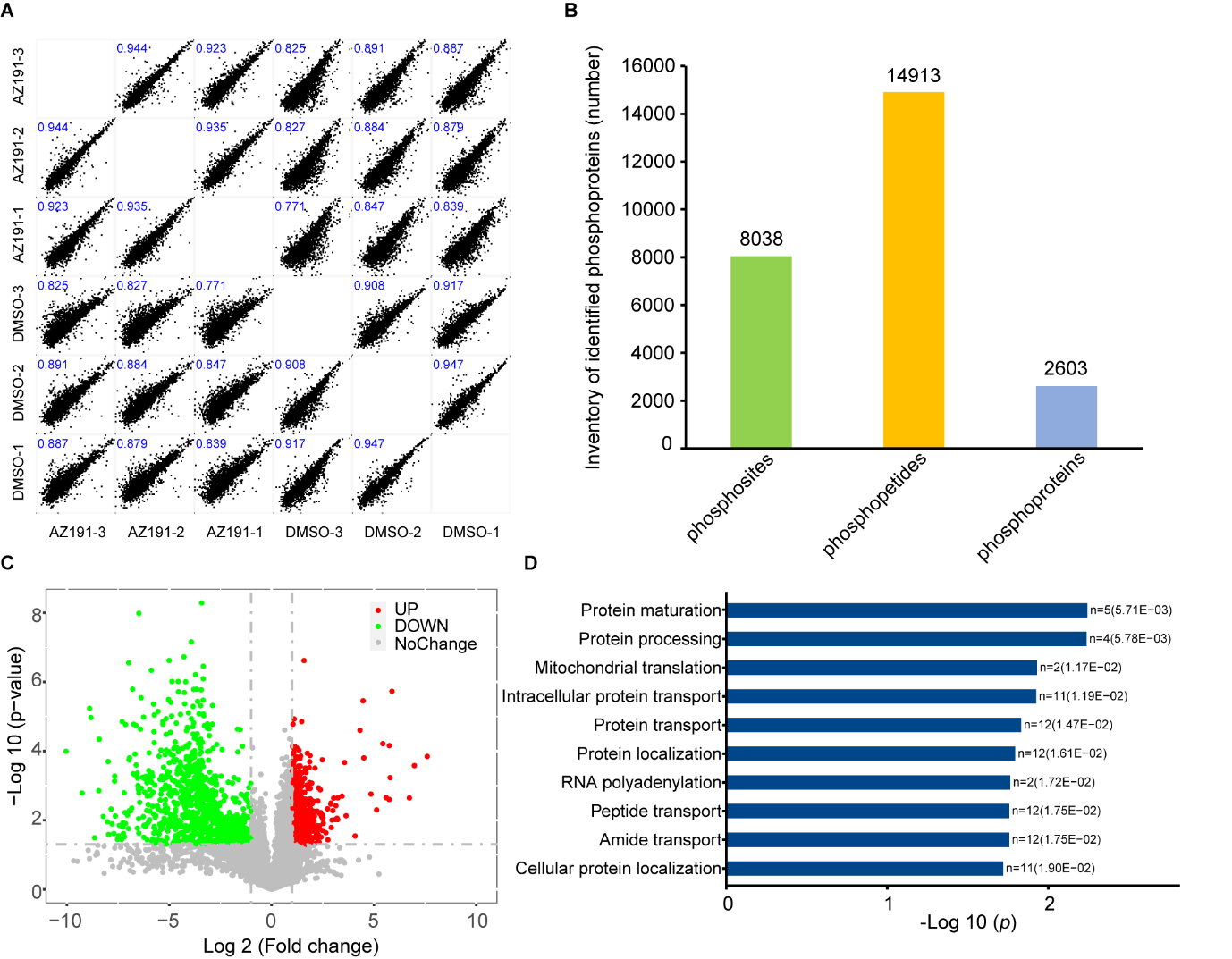


**Figure S2:** Functional enrichment analysis of differentially down-regulated phosphoproteins. (A) The quality assessment of the phosphorylation sequencing result. The values of Pairwise Pearson's correlation coefficients, shown with the blue text, between samples of same treatment were greater than 0.9, which suggests the high reproducibility (R > 0.9) of the phosphoproteomics data. (B) Total number of phosphosites, phosphopeptides, and phosphoproteins detected in phosphoproteomics. (C) Volcano plot of the DMSO/AZ191 group. The standard of fold change > 2 or < 0.5 and a P value < 0.05 were established. (D) GO enrichment of downregulated phosphoproteins.
