# Supplementary material for: DYRK1-mediated phosphorylation of endocytic components is required for extracellular lumen expansion in ascidian notochord
Source: Biol Res. 2023 Mar 11;56:10. doi: 10.1186/s40659-023-00422-9 (PMC10007804; doi:10.1186/s40659-023-00422-9)
Supplement: Supplementary file 3 — Additional file 3: Figure S3. The interaction of DYRK1 with endophilin. (A) Identification of the binding fragment of endophilin with DYRK1. These results showed that the 28 residues at N-terminal of endophilin were required for the interaction of endophilin with DYRK1. (B–C) Endophilin colocalized with DYRK1 in notochord cell membrane and cytoplasm. Plot Profile of ImageJ software was used for colocalization analysis of tdtomato and eGFP signals. The variation trend of fluorescence intensity along the horizontal direction of the white dash line box region in B was showed in C. The red and green lines in C represent the variation trend of tdtomato and eGFP fluorescence intensity, respectively. The trends of red and green curve basically coincident suggest the colocalization of tdtomato and eGFP signals on the cell membrane and in the cytoplasm. The X-axis of C refers to the length in the horizontal direction of white dash line box. Scale bar 20 μm. [file 40659_2023_422_MOESM3_ESM.docx]

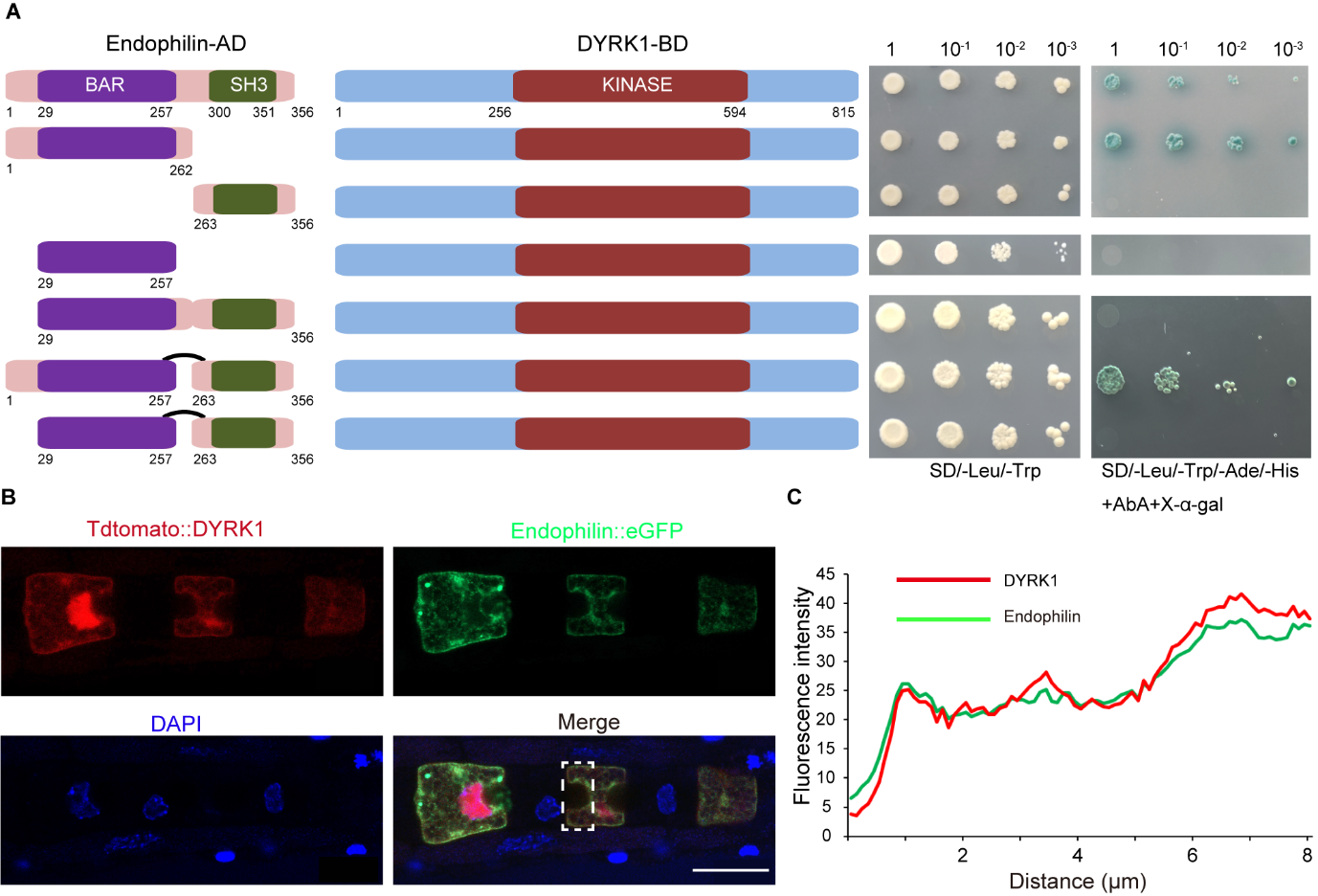


**Figure S3:** The interaction of DYRK1 with endophilin. (A) Identification of the binding fragment of endophilin with DYRK1. These results showed that the 28 residues at N-terminal of endophilin were required for the interaction of endophilin with DYRK1. (B–C) Endophilin colocalized with DYRK1 in notochord cell membrane and cytoplasm. Plot Profile of ImageJ software was used for colocalization analysis of tdtomato and eGFP signals. The variation trend of fluorescence intensity along the horizontal direction of the white dash line box region in B was showed in C. The red and green lines in C represent the variation trend of tdtomato and eGFP fluorescence intensity, respectively. The trends of red and green curve basically coincident suggest the colocalization of tdtomato and eGFP signals on the cell membrane and in the cytoplasm. The X-axis of C refers to the length in the horizontal direction of white dash line box. Scale bar 20 μm.
